# Reorganization of early visual cortex functional connectivity following selective peripheral and central visual loss

**DOI:** 10.1038/srep43223

**Published:** 2017-02-24

**Authors:** Norman Sabbah, Nicolae Sanda, Colas N. Authié, Saddek Mohand-Saïd, José-Alain Sahel, Christophe Habas, Amir Amedi, Avinoam B. Safran

**Affiliations:** 1 Sorbonne Universités, UPMC Université Paris 06, UMR S968, Institut de la Vision, Paris, F-75012, France; 2 INSERM, U968, Institut de la Vision, Paris, F-75012, France; 3 CNRS, UMR 7210, Institut de la Vision, Paris, F-75012, France; 4 Centre d’investigation clinique, Centre Hospitalier National d’Ophtalmologie des Quinze-Vingts, INSERM-DGOS CIC 1423, Paris, F-75012, France; 5 Service de neurologie, Hôpital Foch, Suresnes, France; 6 Institute of Ophthalmology, University College of London, United Kingdom; 7 Fondation Ophtalmologique Adolphe de Rothschild, Paris, France; 8 Department of Ophthalmology, The University of Pittsburgh School of Medicine, Pittsburgh, PA 15213, US; 9 Centre de neuroimagerie, Centre Hospitalier National d’Ophtalmologie des Quinze-Vingts, Paris, F-75012, France; 10 Department of Medical Neurobiology, The Institute for Medical Research Israel-Canada, Faculty of Medicine, The Hebrew University of Jerusalem, Jerusalem 91220, Israel; 11 The Edmond and Lily Safra Center for Brain Sciences (ELSC), The Hebrew University of Jerusalem, Jerusalem 91220, Israel; 12 The Cognitive Science Program, The Hebrew University of Jerusalem, Jerusalem 91220, Israel; 13 Department of Clinical Neurosciences, Geneva University School of Medicine, Geneva, Switzerland

## Abstract

Behavioral alterations emerging after central or peripheral vision loss suggest that cerebral reorganization occurs for both the afferented and deafferented early visual cortex (EVC). We explored the functional reorganization of the central and peripheral EVC following visual field defects specifically affecting central or peripheral vision. Compared to normally sighted, afferented central and peripheral EVC enhance their functional connectivity with areas involved in visual processing, whereas deafferented central and peripheral EVC increase their functional connectivity with more remote regions. The connectivity pattern of afferented EVC suggests adaptive changes that might enhance the visual processing capacity whereas the connectivity pattern of deafferented EVC may reflect the involvement of these regions in high-order mechanisms. Characterizing and understanding the plastic changes induced by these visual defects is essential for any attempt to develop efficient rehabilitation strategies.

The macular and peripheral parts of the retina constitute an intricate functional unit. Their combined input is responsible for the impression of a homogenous image over the entire visual field. This functional unity is not present from birth since ontogenically, the central and peripheral parts of the retina mature sequentially. Unlike the periphery, the central retina is immature at birth and only develops completely years later^1^,^2^,^3^. After the visual function matures, damage to the central or peripheral retina impairs not only its specific functions related to the affected region, but also lessens the performance of the other retina^4^. How the brain behaves and potentially adapts to this challenge remains unclear. Nevertheless, a number of potential response mechanisms have been suggested: (1) the remaining afferented visual cortex tunes-up its processing capacity and compensates to a certain extent for the limited retinal input, whereas the deafferented visual cortex might (2) rewire and receive sensory input from the spared retina and end up treating roughly the same type of information as the afferented visual cortex; (3) divert its processing capacity to specific higher-order functions or multisensory processing; (4) supply the rest of the brain with meaningless input generated from aberrant intrinsic activity.

Adaptive strategies such as the eccentric fixation employed in the case of central visual field defects induce proportional functional changes in the peripheral early visual cortex (EVC)^5^,^6^, thus providing some support for the first hypothesis that the residual afferent visual cortex reorganizes to compensate for the loss in sensory input. In support of the second, rewiring hypothesis, Morland^7^ and Baseler^8^ found that in rod monochromats, deafferented regions of the visual cortex respond to visual stimulation of the functional retina, but that these populations present a differently organized visual system and an abnormal foveal structure^9^ due to the congenital absence of cones. In acquired visual field defects a similar reorganization was reported^10^,^11^, but later challenged^12^. Other authors^13^,^14^,^15^ reported that adults with conditions inducing either central or peripheral field defects only exhibited task-related activation of the deafferented regions of the visual cortex. This led to the third hypothesis of another type of reorganization in which the sensory-deprived visual regions contribute to higher-order mechanisms such as attention or mental imagery^13^,^14^,^15^ or intervene in multisensory processing^16^. The occurrence of visual hallucinations (i.e. the Charles Bonnet syndrome) following both central and peripheral visual loss and their induction through blindfolding in the normally-sighted advocate for the presence of aberrant intrinsic activity in sensory deprivation (the fourth hypothesis)^17^,^18^,^19^. Thus overall, the literature on the reorganization of visual cortex subsequent to partial or total visual loss remains fraught with controversy. In previous studies, factors such as the limited number of participants^10^,^11^,^12^,^13^,^14^,^15^ and/or heterogeneity in the extent of visual field defects in the samples^10^,^11^,^12^,^13^ may have contributed to these divergent results and preclude comparisons between the functional reorganization induced by central and peripheral visual loss. To avoid these obstacles, samples must consist of subjects with comparable, converse visual field defects. In this study, we selected participants suffering from a condition that induces progressive visual loss in either the central retina; i.e., Stargardt macular dystrophy, or the peripheral retina; i.e. retinitis pigmentosa and whose visual field defects met the selection criteria for our experiments.

Stargardt macular dystrophy (SMD) is a well-documented bilateral, inherited retinal disorder that induces well-circumscribed, central visual defects^20^,^21^. In its advanced stages, patients affected by this hereditary cone-rod dystrophy end up losing macular vision and in daily life can only rely on their residual peripheral vision. They are able to orient and navigate, but are markedly impaired for object or face identification and reading^22^,^23^. In contrast, retinitis pigmentosa - a rod-cone dystrophy - is a disorder that primarily affects the peripheral retina, causes progressive bilateral constriction of the visual field and eventually, in its most advanced stages, leads to complete blindness^20^. In the “tunnel vision stage” (RPTV), when the macular function is still preserved, these patients are able to correctly analyze relatively small images but experience difficulties in spatial orientation and scene perception^24^,^25^,^26^.

We explored the changes induced by partial visual loss by analyzing resting-state functional connectivity (rs-FC), a method that places few demands on patients since they perform no task during scan acquisition. Resting-state fluctuations are well-organized into networks previously identified in a range of cognitive tasks^27^,^28^,^29^,^30^,^31^,^32^,^33^,^34^,^35^,^36^, and are of particular interest when attempting to detect interactions between early visual areas, higher order visual areas, and non-visual brain regions both in normal and altered visual function^37^,^38^,^39^. To selectively explore the resting-state FC of central and peripheral early visual cortex, we employed a partial correlation method^39^,^40^. This method is used extensively as a research tool because it is sufficiently sensitive to recover retinotopic or mototopic maps from the resting-state both in the sighted^39^,^40^,^41^,^42^ and the blind^38^,^39^,^43^. It constitutes a highly useful technique when aiming for a global view of these interactions and a detailed map of the background brain FC, a crucial first step in exploring the brain’s response to visual loss. However, to the best of our knowledge, only a few studies have explored the rs-FC of EVC in the visually impaired^44^,^45^, but none have differentiated between its central and peripheral regions^44^,^45^. Given the nature of these patients’ deprivation, it is clearly worth inquiring whether these FC changes are unique to the visual system. Therefore, as a control, we also investigated the possible FC changes in another topographic system, the somatosensory system.

## Materials and Methods

### Subjects and ethics

Subjects were recruited and assigned to the three groups as follows:

- Twelve Stargardt macular dystrophy (SMD) subjects (six women and six men; all subjects were right-handed), presenting a central scotoma, 10–20 degrees in diameter (as evaluated by Goldmann III/4 kinetic perimetry), without foveal sparing, and best-corrected visual acuity equal or superior to 20/400 (measured by EDTRS charts). Ages ranged from 18 to 58 years (average: 38.4, median: 39).
- Twelve retinitis pigmentosa tunnel vision (RPTV) subjects (six women and six men; nine subjects were right-handed), presenting a central residual visual field limited to a 10–20 degree diameter (as evaluated by Goldmann III/4 kinetic perimetry), and best-corrected visual acuity equal or superior to 20/40 (measured by EDTRS charts). Ages ranged from 18 to 62 years (average: 41.8, median: 40.0).
- Fourteen normally sighted controls (seven women and seven men; 14 subjects were right-handed), with normal routine ophthalmological examinations. Ages ranged from 18 to 59 years (average: 41.6, median: 41.0).

Subjects were matched for age, and no subject had any reported neurological or psychiatric diseases (see Table 1). The Ethics Committee (Comité de protection des personnes, Ile de France V, and Agence Nationale de Sécurité du Médicament et des Produits de Santé) approved the experimental protocol (number 12873), and all subjects gave their written informed consent before participating. All methods were performed in accordance with the relevant guidelines and regulations.

### Functional imaging

fMRI was conducted with a whole-body 3T clinical imager (Sigma Horizon) using an eight-channel head coil. In each scanning sequence, 32 contiguous axial T2*-weighted gradient-echo echo-planar images (TE/TR, 93 ms/3000 ms; FOV, 240 × 240 mm; matrix, 64 × 64; voxel size, 3.75 × 3.75 × 4 mm; thickness, 4 mm; interslice spacing, 0 mm; NEX, 1) were recorded to encompass the entire brain. 180 volumes were acquired including 4 “dummy” volumes obtained at the start of the session. Scan duration was 9.25 minutes for the EPI sequence. Off-line, T2*-weighted images were co-registered and overlaid on the corresponding anatomic T1-weighted gradient-echo images (TE/TR/flip angle, 3.9 ms/9.5 ms/20°; FOV, 25.6 × 25.6 mm; matrix, 512 × 512; source voxel size, 1.2 × 0.5 × 0.5 mm converted to 1 × 1 × 1 mm; thickness, 1.2 mm; interslice spacing, 1.2 mm). During the scan, subjects were supine in the MRI scanner and wore earplugs to compensate for the noisy environment. Subjects were instructed to keep their eyes closed. No explicit task was required.

### fMRI preprocessing

fMRI data were preprocessed using the BrainVoyager QX 2.8 software package (Brain Innovation, Maastricht, Netherlands) and complementary software written in MATLAB R2009a (The MathWorks, USA). Preprocessing of functional scans successively included: 3D motion correction (no head motion exceeded 2 mm/2 degrees in any of the six movement directions i.e. X, Y, Z translations, X, Y, Z rotations), slice-time correction, band-pass filtering between 0.01 and 0.1 Hz, voxel-to-voxel linear regression^46^ of spurious signals from the white matter and ventricle regions anatomically defined for each subject, normalization in the Talairach coordinate system in the volume^47^, and spatial smoothing with a 6 mm Gaussian filter kernel full-width-at-half-maximum. We did not include global signal removing to avoid the possible introduction of false negative correlations^48^. For the between-group comparison, we tested and found no significant difference in the maximum head movement^49^ between the three groups (SMD: 0.29 ± 0.27 mm; RPTV: 0.16 ± 0.08 mm; sighted controls: 0.19 ± 0.18 mm; ANOVA F (2, 35) = 1.5; p = 0.23). We also performed a GLM analysis including the head movement predictors (control analysis; see Supplementary Figures S1, S2, S3 and S4). Including movement predictors in the GLM did not change the main results.

### External functional localizers

External functional localizers were used to define the seed region-of-interest (ROI) from visual localizers (central EVC, peripheral EVC) in the volume. Each of these localizers was extracted from a group of normally sighted controls and analyzed in normalized Talairach space using a random effect GLM which enabled generalization of the findings to the population (see below; Friston *et al*. 1999).

To define the primary visual cortex seeds, a separate population of thirteen control subjects (seven women; six male; aged 22–35) was scanned in a standard phase-encoded retinotopic mapping experiment, using ring (eccentricity mapping) and wedge (polar mapping) stimuli^39^,^50^ delivered during two separate sessions. The stimuli were projected via an LCD screen positioned over the subject’s forehead and watched through a tilted mirror. In the first session an annulus was projected, expanding in 30 seconds from 0° to 34° of the subject’s visual field; the procedure was repeated ten times. The second session included a wedge stimulus with a 22.5° polar angle that rotated around the fixation point; each cycle was completed in 30 seconds, and repeated 20 times. Both the annulus in the first session and the wedge in the second session contained a flickering (6 Hz) radial checkerboard pattern with respect to standard retinotopic procedures^50^ for the delineation of field maps. In both cases, there was a 30 second mute period before and after the visual stream for baseline. Group phase analysis was conducted on the two sessions^51^ resulting in group maps of eccentricity and angle mapping. Angle mapping was then used to define the borders of EVC, and the two maps were used to segregate it according to eccentricity (center or periphery of the visual field). The central EVC was considered under an eccentricity of 5 degrees and the peripheral EVC higher than 15 degrees.

For supplementary ROI analyses, we also used external functional localizers from previous studies by our team^40^,^42^,^52^ i.e. left hemisphere S1 lip and foot areas.

To validate the localizer definitions, we also ran a control analysis (see supplementary Figure S7) at the individual subject level to test for the time course correlations between seed regions defined based on external functional localizers and seed regions defined based on anatomical localizers (as defined by 7 mm spheres on the posterior and anterior portions of the calcarine sulcus of each subject).

### Seed ROI definition and analysis

1. A partial correlation analysis^39^,^53^ was conducted as follows. For each subject, a seed region of interest (ROI) based on external functional localizers (*e.g.,* the central EVC) served to extract and z-transform the blood-oxygen-level dependent time course of this region in volume^54^ while regressing out the time course of the complementary part of the visual field component (*e.g.,* the peripheral EVC) to suppress the shared variance and conserve only the unique variance corresponding to the ROI predictor.
2. A random-effect analysis based on the general linear model (GLM)^55^ was conducted for each group. This pair-wise correlation analysis led to four maps for each group: (a) the central EVC (regressed peripheral EVC), (b) the peripheral EVC (regressed central EVC), (c) the left S1 – lip area (regressed S1 – foot area) and (d) the left S1 – foot area (regressed S1 – lip area) with a significance level of p < 0.05, corrected for multiple comparisons using cluster-size thresholding^39^,^56^, implemented in BrainVoyager using the Monte Carlo simulation approach (1000 iterations). This method directly takes into account the data contiguity of neighboring voxels and corrects for the false-positive rate of continuous clusters (a set-level statistical inference correction; corrected to p < 0.05).
3. For each patient group, we also tested the correlations between the following variables (*i*) the age at disease onset and, (*ii*) the duration of the visual deficit (Pearson Product-Moment Correlation, corrected for false discovery rate (FDR) for multiple comparisons) and the FC patterns found in the between-group maps (pairs of regions significantly connected; *e.g.* peripheral EVC and lingual gyrus).
4. To test for the main effect, a random-effect ANOVA was run on all the subjects with a between-subject factor enabling separation between groups with a significance level of p < 0.05, corrected for multiple comparisons using cluster-size thresholding.
5. To establish contrasts between groups, second-level analyses (post-hoc) were performed.

To summarize, an ROI-based functional connectivity analysis applied to the brain resting-state fMRI data was conducted on all groups. We used data from the seeded regions to establish resting-state functional connectivity maps intra-group. We then ran an ANOVA on all the subjects and post-hoc analysis to determine differences between groups in volume. The maps were then converted and displayed on the surface (data integrated in depth along vertex normals from −1 mm to 3 mm), enabling a complete view of the brain.

## Results

All the results presented below were statistically significant (p < 0.05; corrected for multiple comparisons).

### Intra-group analysis - Functional connectivity seeded from the central EVC

**Figure 1:**
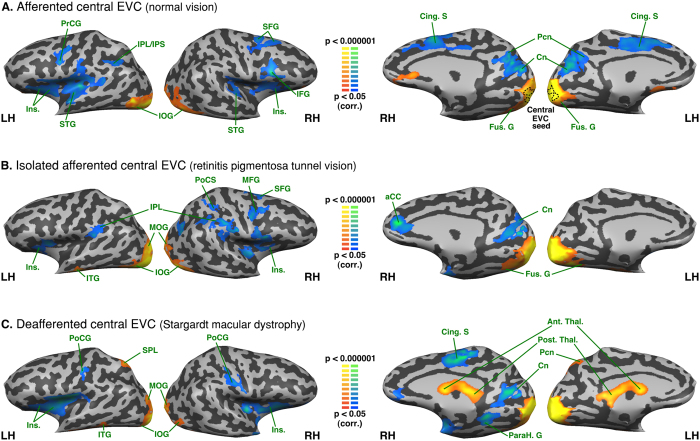
Intra-group analysis of functional connectivity seeded from the central EVC (peripheral EVC regressed). The maps are shown in mesh for the (**A**) afferented central EVC (normal vision); * the dotted line on the medial aspect of the brain represents the seed region (central EVC) (**B**) isolately afferented central EVC (retinitis pigmentosa tunnel vision) (**C**) deafferented central EVC (Stargardt macular dystrophy). Yellow-orange depicts areas of higher positive functional connectivity for each group, and green-blue higher negative functional connectivity. LH: left hemisphere, RH: right hemisphere. Ant. Thal.: anterior thalamus; Cing. S: cingulate sulcus; Cn: cuneus; Fus. G: fusiform gyrus; IFG: inferior frontal gyrus; Ins.: Insula; IOG: inferior occipital gyrus; IPL: inferior parietal lobule; IPS: intraparietal sulcus; ITG; inferior temporal gyrus; Ling. G: lingual gyrus; MFG: middle frontal gyrus; MOG: middle occipital gyrus; ParaH. G: parahippocampal gyrus; Pcn: precuneus; Post.Thal.: posterior thalamus; PrCG: precentral gyrus; PoCS: postcentral sulcus; SFG: superior frontal gyrus; SPL: superior parietal lobule.

*Afferented central EVC* in the normally sighted exhibited a positive FC with the bilateral inferior and middle occipital and bilateral posterior part of the lingual gyri, bilateral orbitofrontal cortex but a negative FC with the bilateral inferior frontal gyrus, insula, cuneus, parieto-occipital sulcus, precuneus, cingulate sulcus, superior temporal and frontal gyrus, left intraparietal sulcus, inferior parietal lobule, precentral gyrus and right superior frontal sulcus (see Fig. 1A).

*Isolated afferented central EVC* in the RPTV exhibited a positive FC with the bilateral posterior part of the lingual gyrus, bilateral inferior and middle occipital and posterior fusiform gyri. It exhibited a negative FC with the bilateral inferior parietal lobule (supramarginal gyrus), insula, right anterior cingulate gyrus and right cuneus, right intraparietal sulcus, precentral sulcus and the posterior part of superior, middle and inferior frontal gyri (see Fig. 1B).

*Deafferented central EVC* in the SMD exhibited a positive FC with the bilateral posterior part of the lingual gyrus, bilateral inferior occipital gyrus, middle occipital gyrus, left superior parietal lobule, precuneus, bilateral anterior and posterior thalami. It exhibited a negative FC with the bilateral insula and postcentral gyrus, right posterior part of cingulate sulcus and the medial part of the precentral gyrus, right cuneus, anterior part of the lingual and parahippocampal gyri (see Fig. 1C).

There were no significant correlations for any group between either the age at disease onset or the duration of the visual deficit, and the FC of the central EVC with any other significant regions in the intra-group maps (Pearson correlations, FDR corrected; all ps > 0.05).

### Intra-group analysis - Functional connectivity seeded from the peripheral EVC

**Figure 2:**
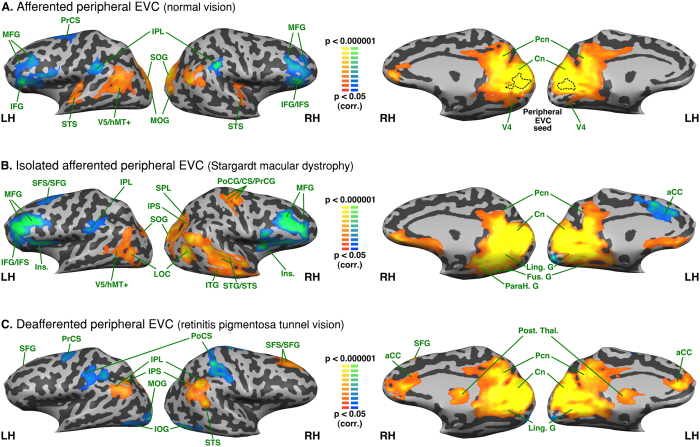
Intra-group analysis of functional connectivity seeded from the peripheral EVC (central EVC regressed). The maps are shown in mesh for the (**A**) afferented peripheral EVC (normal vision); * the dotted line on the median aspect of the brain represents the seed region (peripheral EVC) (**B**) isolated afferented peripheral EVC (Stargardt macular dystrophy) (**C**) deafferented peripheral EVC (retinitis pigmentosa tunnel vision). Yellow-orange depicts areas of higher positive functional connectivity for each group, and green-blue higher negative functional connectivity. LH: left hemisphere, RH: right hemisphere. aCC: anterior cingulate cortex; CS: central sulcus; Cn: cuneus; Fus. G: fusiform gyrus; IFG: inferior frontal gyrus; IFS: inferior frontal sulcus; Ins.: insula; IOG: inferior occipital gyrus; IPL: inferior parietal lobule; IPS: intraparietal sulcus; ITG: inferior temporal gyrus; ITS: inferior temporal sulcus; Ling. G: lingual gyrus; LOC: lateral occipital complex; MFG: middle frontal gyrus; MOG: middle occipital gyrus; Pcn: precuneus; PrCS: precentral sulcus; Post.Thal.: posterior thalamus; PrCG: precentral gyrus; PoCS: postcentral sulcus; SFG: superior frontal gyrus; SFS: superior frontal sulcus; SOG: superior occipital gyrus; SPL: superior parietal lobule STG: superior temporal gyrus; STS: superior temporal sulcus; V5/hMT+: V5/human MT+.

*Afferented peripheral EVC* in the normally sighted exhibited positive FC with the bilateral cuneus, precuneus, posterior cingulate, lingual, parahippocampal, fusiform, superior and middle occipital gyri, posterior middle temporal gyrus and superior temporal sulcus, anterior superior temporal gyrus and right orbitofrontal and anterior cingulate cortex. It exhibited a negative FC with the bilateral inferior and middle frontal gyri, precentral sulcus, inferior frontal sulcus, inferior parietal lobule (supramarginal gyrus) and left superior frontal gyrus (see Fig. 2A).

Isolated a*fferented peripheral EVC* in SMD exhibited a positive FC with the bilateral cuneus, lingual gyrus, parahippocampal gyrus, fusiform gyrus, precuneus, posterior cingulate, superior occipital gyrus, right middle occipital gyrus, bilateral inferior parietal lobule, right posterior superior parietal lobule and intraparietal sulcus, bilateral posterior middle temporal gyrus and superior temporal sulcus, bilateral lateral occipital complex, right superior, middle and temporal gyri and right superior and inferior temporal sulci, right pre and post central gyri, right central sulcus and bilateral orbitofrontal cortex. It exhibited a negative FC with the bilateral insula, inferior and middle frontal gyri, inferior and superior frontal sulci, right superior frontal gyrus and left anterior cingulate cortex (see Fig. 2B).

*Deafferented peripheral EVC* in RPTV exhibited a positive FC with the bilateral cuneus, lingual and parahippocampal gyri, precuneus, posterior cingulate, anterior cingulate, inferior parietal lobule, posterior thalamus, right posterior superior temporal sulcus and superior frontal gyrus and sulcus. It exhibited a negative FC with the bilateral inferior occipital gyri, precentral gyri, postcentral sulci, the anterior part of intraparietal sulci and the left middle occipital gyrus (see Fig. 2C).

There were no significant correlations for any group between either the age at disease onset or the duration of the visual deficit, and the FC of peripheral EVC with any other significant regions in the intra-group maps (Pearson correlations, FDR corrected; all ps > 0.05).

### Between group analysis - Functional connectivity differences seeded from the central EVC

**Figure 3:**
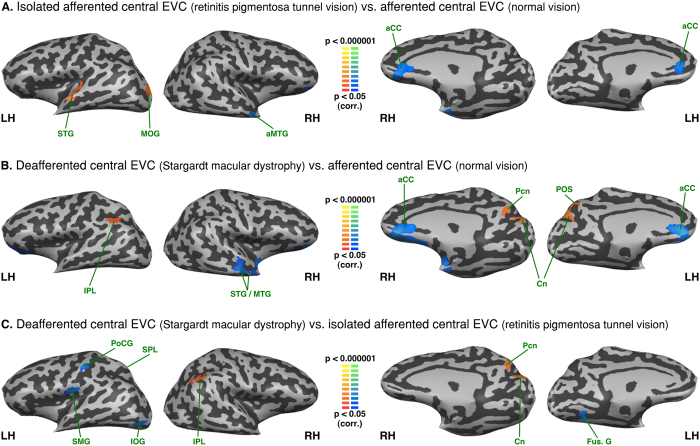
Between-group analysis of functional connectivity seeded from the central EVC (peripheral EVC regressed). The maps are shown in mesh for (**A**) retinitis pigmentosa tunnel vision vs. normal vision (**B**) Stargardt macular dystrophy vs. normal vision (**C**) Stargardt macular dystrophy vs. retinitis pigmentosa tunnel vision (yellow-orange depicts areas of higher positive/lower negative functional connectivity with the central EVC for the first group compared to the second, and green-blue the opposite comparison; LH: left hemisphere, RH: right hemisphere). aCC: anterior cingulated cortex; aMTG: anterior middle temporal gyrus; Cn: cuneus; Fus. G: fusiform gyrus; SPL: superior parietal lobule; IPL: inferior parietal lobule; IOG: inferior occipital gyrus; MOG: middle occipital gyrus; MTG: middle temporal gyrus; Pcn: precuneus; PoCG: postcentral gyrus POS: parieto-occipital sulcus; STG: superior temporal gyrus; SMG: supramarginal gyrus.

As compared to normally sighted, the RPTV subjects showed increased FC of the afferented central EVC with the left middle occipital and left superior temporal gyri. There was also reduced central EVC FC with the anterior part of the right middle temporal gyrus, as well as the bilateral ventral anterior cingulate cortex (see Fig. 3A and Table 2). SMD subjects presented increased FC of the deafferented central EVC with the left inferior parietal lobule, bilateral cuneus, intraparietal sulcus, regions of the dorsal pathway, as well as the right precuneus (see Fig. 3B and Table 2). They also exhibited decreased central EVC FC with the ventral anterior cingulate cortex, the right superior and the middle temporal gyri.

A comparison of the two groups of visually deprived subjects indicated that the SMD subjects presented increased FC between the deafferented central EVC and the right cuneus and precuneus, the right inferior parietal lobule and the left superior parietal lobule (see Fig. 3C and Table 2). RPTV subjects showed increased FC with the left posterior fusiform, the inferior occipital gyri as well as with the left supramarginal gyrus and the postcentral gyrus.

### Between group analysis - Functional connectivity differences seeded from the peripheral EVC

**Figure 4:**
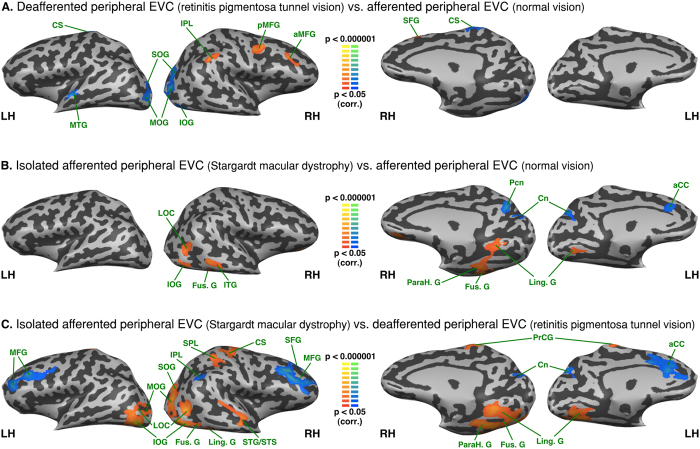
Between-group analysis of functional connectivity seeded from the peripheral EVC (central EVC regressed). The maps are shown in mesh for (**A**) retinitis pigmentosa tunnel vision vs. normal vision (**B**) Stargardt macular dystrophy vs. normal vision (**C**) Stargardt macular dystrophy vs. retinitis pigmentosa tunnel vision (yellow-orange depicts areas of higher positive/lower negative functional connectivity with central EVC for the first group compared to the second, and green-blue the opposite comparison; x, y, z are Talairach coordinates; LH: left hemisphere, RH: right hemisphere). aCC: anterior cingulate cortex; aMFG: anterior middle frontal gyrus; aMTG: anterior middle temporal gyrus; Cn: cuneus; CS: central sulcus; Fus. G: fusiform gyrus; Ling. G: lingual gyrus; LOC: lateral occipital complex; IOG: inferior occipital gyrus; ITG: inferior temporal gyrus; IPL: inferior parietal lobule; MFG: middle frontal gyrus; MOG: middle occipital gyrus; MTG: middle temporal gyrus; ParaH G: parahippocampal gyrus; Pcn: precuneus; pMFG: posterior middle frontal gyrus; PoCG: postcentral gyrus; POS: parieto-occipital sulcus; SFG: superior frontal gyrus; SMG: supramarginal gyrus; SOG: superior occipital gyrus; SPL: superior parietal lobule; STG: superior temporal gyrus; STS: superior temporal sulcus.

As compared to normally sighted, the RPTV group exhibited increased FC with the right inferior parietal lobule, as well as with the posterior and anterior regions of the medial frontal gyrus (see Fig. 4A and Table 2). In contrast to the central EVC FC, the peripheral sensory deprived EVC of RPTV subjects showed decreased FC with the central sulcus left anterior middle temporal gyrus and the middle occipital gyrus as well as with the bilateral superior occipital gyrus, the right middle and the inferior occipital gyrus. The SMD group showed increased FC of their peripheral afferented EVC with regions of the ventral and dorsal pathway such as the right lateral occipital cortex, the bilateral lingual gyri, the right inferior occipital, fusiform, parahippocampal gyri as well as the right inferior temporal gyrus (see Fig. 4B and Table 2). There was decreased FC with the bilateral superior part of the cuneus, the right precuneus and the left dorsal anterior cingulate.

Comparing both groups of visually-deprived subjects showed increased FC of the SMD subjects’ still afferented, peripheral EVC with both ventral and dorsal stream regions – the bilateral middle occipital, inferior occipital gyri, the right superior occipital gyrus, the right lateral occipital cortex, the bilateral lingual and fusiform gyri as well as the right parahippocampal gyrus (see Fig. 4C and Table 2). FC was also increased with the right sensorimotor cortex. By contrast, RPTV subjects had increased FC of their sensory deprived peripheral EVC with the bilateral cuneus on the dorsal pathway, the bilateral middle frontal and the right superior frontal gyri well as with the entire left anterior cingulate cortex.

### Intra-group analysis - Functional connectivity seeded from the left S1 lip area and the left S1 foot area

**Figure 5:**
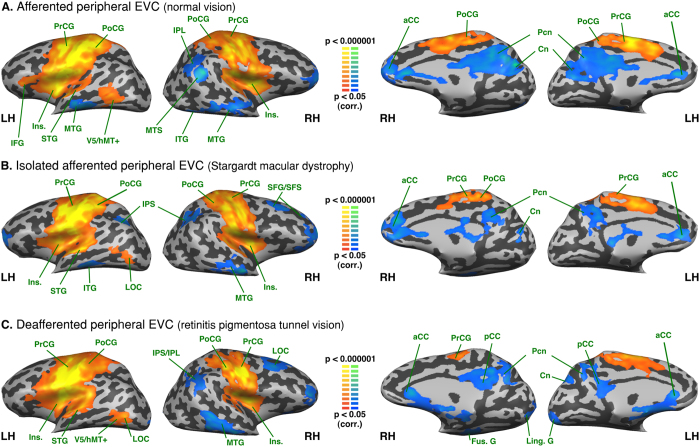
Intra-group analysis of functional connectivity seeded from the left hemisphere S1 lip area (S1 foot area regressed). The maps are shown in mesh for the (**A**) afferented peripheral EVC (normal vision) (**B**) isolated afferented peripheral EVC (Stargardt macular dystrophy) (**C**) deafferented peripheral EVC (retinitis pigmentosa tunnel vision). Yellow-orange depicts areas of higher positive connectivity for each group, and green-blue higher negative connectivity. LH: left hemisphere, RH: right hemisphere. Cn: cuneus; Fus. G: fusiform gyrus; IFG: inferior frontal gyrus; Ins.: Insula; IOG: inferior occipital gyrus; IPL: inferior parietal lobule; IPS: intraparietal sulcus; STG: superior tempral gyrus; ITG: inferior temporal gyrus; Ling. G: lingual gyrus; IFG: inferior frontal gyrus; MOG: middle occipital gyrus; Pcn: precuneus; PrCG: precentral gyrus; PoCG: postcentral gyrus; MTG: middle temporal gyrus; LOC: lateral occipital complex; aCC: anterior cinulate cortex; pCC: posterior cingulate cortex; SFG/SFS: superior frontal gyrus/sulcus.

**Figure 6:**
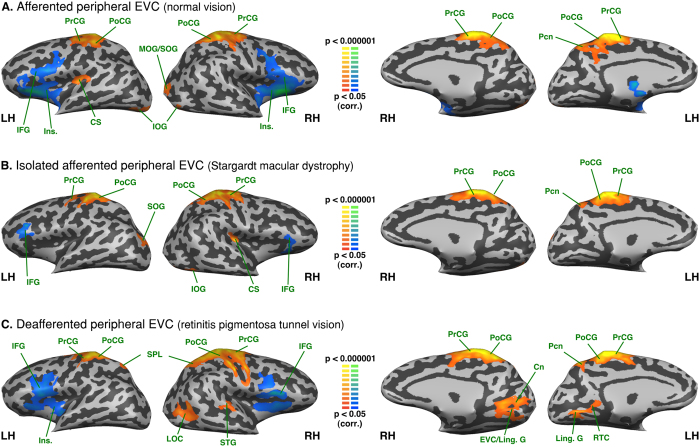
Intra-group analysis of functional connectivity seeded from the left hemisphere S1 foot area (S1 lip area regressed). The maps are shown in mesh for the (**A**) afferented peripheral EVC (normal vision) (**B**) isolated afferented peripheral EVC (Stargardt macular dystrophy) (**C**) deafferented peripheral EVC (retinitis pigmentosa tunnel vision). Yellow-orange depicts areas of higher positive connectivity for each group, and green-blue higher negative connectivity. LH: left hemisphere, RH: right hemisphere. EVC: early visual cortex; Cn: cuneus; RTC: retrosplenial cortex; Fus. G: fusiform gyrus; IFG: inferior frontal gyrus; Ins.: Insula; IOG: inferior occipital gyrus; SPL: superior parietal lobule; IPS: intraparietal sulcus; Ling. G: lingual gyrus; IFG: inferior frontal gyrus; MOG: middle occipital gyrus; SOG: superior occipital gyrus; LS: lateral sulcus; STG: superior temporal gyrus; Pcn: precuneus; PrCG: precentral gyrus; PoCG: postcentral gyrus; MTG: middle temporal gyrus; LOC: lateral occipital complex; aCC: anterior cinulate cortex; pCC: posterior cingulate cortex. CS: central sulcus.

Both S1 respective lip and foot areas were perfectly connected between hemispheres in the three groups (see Figs 5 and 6). The lip area was functionally connected to the bilateral post central gyrus, precentral gyrus, premotor cortex, insula, superior temporal gyrus and the left LOC/V5/hMT+ in all three groups. There was negative FC between the lip area and IPL, anterior cingulate cortex (aCC), anterior MTG, and fronto-orbital cortex, precuneus and cuneus.

The left foot area was functionally connected with bilateral post central gyrus, precentral gyrus, premotor cortex, and precuneus in all three groups. In all groups the left foot area also exhibited negative FC with bilateral IFG and in peripheral visual field defect (RPTV) and in normal vision also with the anterior insula as well. Moreover, the central visual field group (SMD) exhibited increased FC between the left foot area and the left SOG and the right inferior occipital gyrus whereas the peripheral visual field defect group (RPTV) exhibited increased FC with the right peripheral EVC, LOC, superior temporal gyrus and bilateral SPL (see Fig. 6B and C).

### Between group analysis - Functional connectivity seeded from the left S1 lip area and the left S1 foot area

**Figure 7:**
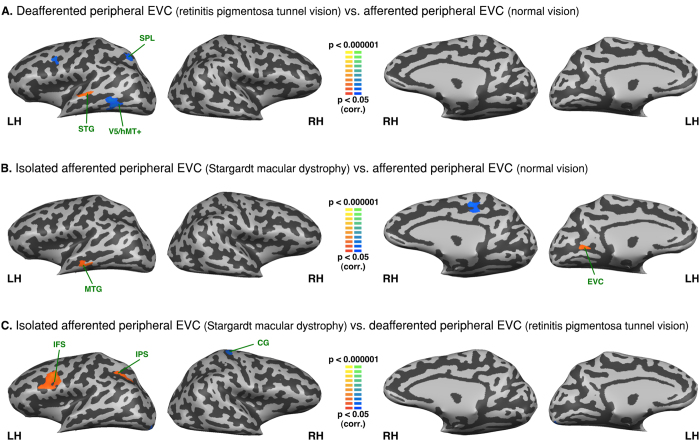
Between-group analysis of functional connectivity seeded from the left hemisphere S1 lip area (S1 foot area regressed). The maps are shown in mesh for (**A**) retinitis pigmentosa tunnel vision vs. normal vision (**B**) Stargardt macular dystrophy vs. normal vision (**C**) Stargardt macular dystrophy vs. retinitis pigmentosa tunnel vision (yellow-orange depicts areas of higher positive/lower negative functional connectivity with S1 lip area for the first group compared to the second, and green-blue the opposite comparison; LH: left hemisphere, RH: right hemisphere). SPL: superior parietal lobule; STG: superior temporal gyrus; MTG: middle temporal gyrus; EVC: early visual cortex; IFS: inferior frontal sulcus; IPS: intraparietal sulcus; CG: central gyrus.

In patients with a peripheral visual field defect compared to normal vision, the left S1 lip area showed increased FC with STG and decreased FC with an area that overlaps V5/hMT+ and EBA as well as SPL (see Fig. 7A). In patients with central visual loss compared to normal vision, there was an increased FC of the lip area with MTG and anterior EVC (see Fig. 7B). In patients with central visual loss compared with patients with peripheral visual loss there is an increased FC of the left lip area with the posterior part of inferior and middle frontal gyri (posterior part of Broca area) and IPS (see Fig. 7C).

**Figure 8:**
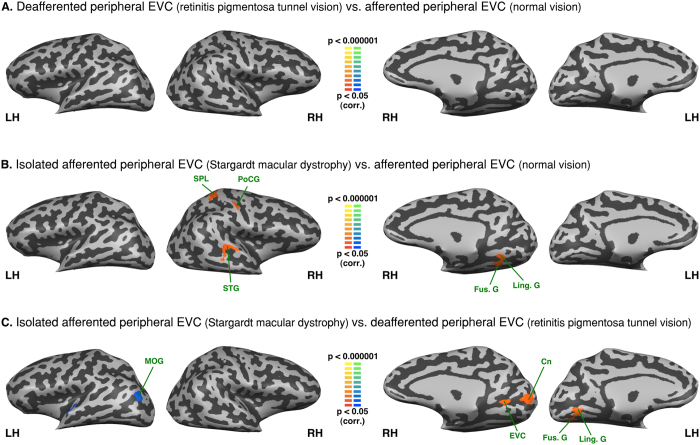
Between-group analysis of functional connectivity seeded from the left hemisphere S1 foot area (S1 lip area regressed). The maps are shown in mesh for (**A**) retinitis pigmentosa tunnel vision vs. normal vision (**B**) Stargardt macular dystrophy vs. normal vision (**C**) Stargardt macular dystrophy vs. retinitis pigmentosa tunnel vision (yellow-orange depicts areas of higher positive/lower negative functional connectivity with S1 foot area for the first group compared to the second, and green-blue the opposite comparison; LH: left hemisphere, RH: right hemisphere). SPL: superior parietal lobule; PoCG: post central gyrus; STG: superior temporal gyrus; Fus. G: fusiform gyrus; Ling. G: lingual gyrus; MOG: middle occipital gyrus; EVC: early visual cortex; Cn: Cuneus.

We found no FC difference for the left foot area between normally sighted and patients with peripheral visual field loss (RPTV) (see Fig. 8A). In patients with central visual field loss (SMD) compared to the normally sighted, there was increased FC with the right posterior superior temporal gyrus, SPL, postcentral gyrus, fusiform and lingual gyri (see Fig. 8B). Compared to central visual field loss (SMD), in peripheral visual field loss, the left foot area exhibited increased FC with the right anterior EVC, cuneus and left lingual and fusiform gyri and decreased FC with left MOG (see Fig. 8C).

**Figure 9:**
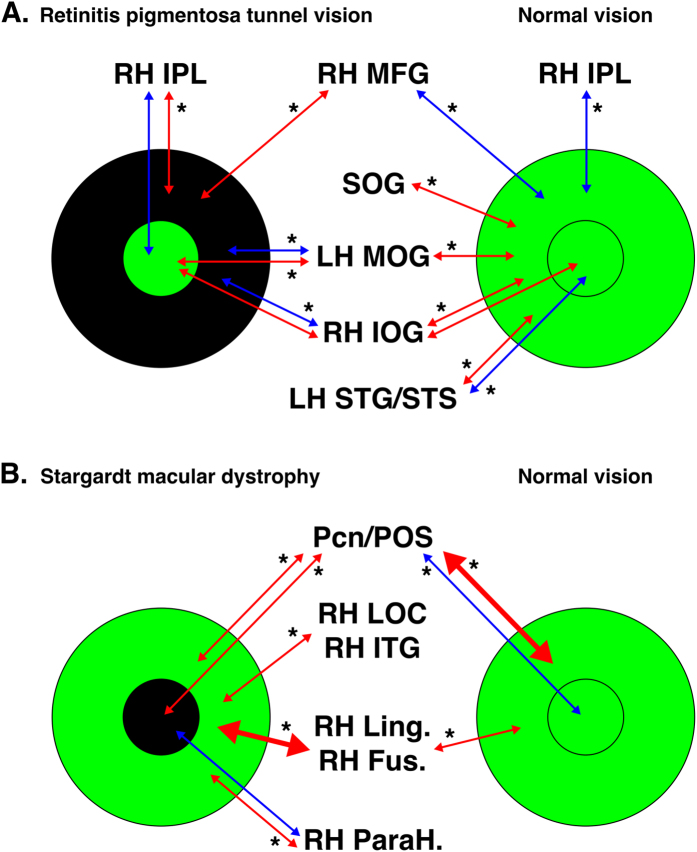
Summary of the main results of the FC analysis seeding peripheral and central EVC. The main FC results are shown for the comparison between (**A**). retinitis pigmentosa tunnel vision or (**B)**. Stargardt macular dystrophy and normal vision. Small circles indicate regions of the EVC coding for the central projection of the retina, large circles indicate regions of the EVC coding for the peripheral projection of the retina. Green depicts afferented areas of the EVC. Black depicts deafferented areas of the EVC. Red arrows indicate positive FC between areas at the intra-group level and blue arrows negative FC between areas at the intra-group level. The arrow thickness indicates the level of FC at the intra-group level. Stars indicate statistical significance at p < 0.05 for the FC between groups. LH: left hemisphere, RH: right hemisphere. MFG: middle frontal gyrus; STG/STS: Superior temporal gyrus/sulcus; Cn: cuneus; Fus. G: fusiform gyrus; Ling. G: lingual gyrus; LOC: lateral occipital complex; IOG: inferior occipital gyrus; ITG: inferior temporal gyrus; IPL: inferior parietal lobule; MFG: middle frontal gyrus; MOG: middle occipital gyrus; ParaH G: parahippocampal gyrus; Pcn: precuneus; POS: parieto-occipital sulcus; SOG: superior occipital gyrus.

The main results were summarized in Fig. 9.

## Discussion

SMD and RPTV models were implemented to evaluate the effects of central and visual loss on the FC of the EVC. The five main findings can be summarized as follows: (1) in both patient groups, deafferented EVC exhibited a different FC pattern than the corresponding regions in normally sighted, suggesting that either the central or peripheral EVC could reorganize after deafferentation but more interestingly (2) we also found a different FC pattern in the patients’ EVC that still received the residual visual input, implying that such reorganization also impacts afferented visual areas; (3) we found more changes in FC to peripheral than to central EVC (in both afferented and deafferended states); (4) compared to normal vision, the patients’ afferented central or peripheral EVC demonstrated increased FC with many areas involved in visual processing whereas the deafferented central or peripheral EVC showed increased FC with remote regions; (5) The contrast between deafferented and isolated afferented early visual cortex revealed patterns of FC differences that were similar, but more complex compared to those observed in comparison to normally afferented visual cortex.

### The functional connectivity of the normally afferented central and peripheral early visual cortex

In the normally sighted, the central and peripheral early visual cortex exhibited a positive FC with areas of the dorsal and ventral stream. The positive FC of the central and peripheral EVC was roughly similar to previous descriptions of the entire EVC connectivity with the exception of the primary sensorimotor cortex^37^. However, we found a weak positive FC between the early visual cortex (central and peripheral) and the primary sensorimotor cortex (mostly in the right hemisphere) that did not survive the correction for multiple comparisons (data not shown).

### FC differences between isolated afferented and normally afferented EVC

Compared to the subjects with normal vision, the afferented central EVC in RPTV exhibited increased FC with the left MOG and STG, whereas their deafferented EVC periphery showed decreased FC with these same regions. Interestingly, in normal vision these regions had stronger canonical connections with the peripheral EVC as found in previous studies (see Fig. 2)^57^,^58^,^59^. It is conceivable that the increased FC of the central EVC with the left MOG and STS/STG, which are involved in space, scene processing and multisensory integration, reflects an adaptive process to partially compensate for the loss of peripheral vision^60^,^61^,^62^,^63^.

Compared to the subjects with normal vision, the afferented peripheral EVC in SMD subjects showed an increased FC with areas of the ventral stream (i.e., the left lingual and inferior occipital gyri, the bilateral fusiform gyrus, the right parahippocampal gyrus) and associated functional areas (i.e., the right lateral occipital complex). Most of these regions are involved in the face perception network^64^,^65^ and even predict performance in multiple face-processing tasks^66^. It is worth noting that in normal vision, face perception is associated with center-biased rather than peripheral-biased representations^64^,^67^. Hence, the increased FC between isolated afferented peripheral EVC and the ventral stream areas in SMD subjects (as compared to the other groups) may be indicative of a compensatory mechanism for the loss of central vision. Similar results were obtained in an fMRI study exploring the effects of simulated central scotoma on face recognition^68^. Moreover, developmental data on the early ability of neonates to process faces^69^ although they have an immature fovea and low visual acuity^1^ lend additional support to this hypothesis.

### FC differences between afferented and deafferented EVC

Compared to the normally sighted, the deafferented peripheral EVC in RPTV group exhibited increased FC with the IPL/IPS and MFG. Interestingly, the isolated afferented central EVC showed decreased FC with the same region. IPS/IPL plays a role in top-down control, attention, multisensory integration, visuomotor coordinate transformation and task-demand in normal subjects^58^,^70^,^71^,^72^. Therefore, the increased FC between the RPTV deafferented peripheral EVC with IPS and other areas involved in eye movements (FEF, SEF), multisensory integration, visuomotor coordinate transformation (IPL) and pre-motor control and planning (middle frontal gyrus) presumably reflects adaptive mechanisms to peripheral vision loss.

Compared to the normally afferented (sighted) and isolated afferented central EVC (RPTV), the deafferented central EVC of the SMD exhibited increased FC with the cuneus, the parieto-occipital sulcus (POS) and the precuneus. The participation of these regions in switching attention, orientation selectivity and visuomotor processing^73^ raises questions about to their possible role in the development of a surrogate overt attention network in subjects lacking central vision. This remains true as well at the intragroup level given the positive FC of the deafferented central EVC not only with the precuneus but also with the MOG (see Fig. 1C), which is involved in space and scene processing^63^. In the normally sighted EVC, the precuneus and MOG exhibit strong coactivation in visual imagery tasks^74^ suggesting that the FC changes we observed in the deafferented central EVC might share common mechanisms with mental imagery^74^. Interestingly, in contrast to the deafferented central EVC, the isolated afferented peripheral EVC of SMD patients exhibited decreased FC with the cuneus, the POS and the precuneus (see Fig. 4B) possibly reflecting a buffering mechanism to prevent noise and errors^75^.

At the intra-group level, both the deafferented central (SMD) and peripheral (RPTV) EVC showed a positive FC with the posterior thalamus/pulvinar (non- significant difference when compared to normally sighted). The pulvinar is involved in attentional processes and is thought to play a fundamental role in cortico-cortical communication^76^. Sensory deprivation imbalances the geniculate and cortical input^13^,^15^ and therefore, the positive FC between the visually deafferented early visual cortex and the pulvinar might unmask preexisting cortico-thalamo-cortical signals for top-down control. Activation found by previous studies in the V1 lesion projection zone (LPZ) during visual stimulus-related judgments but not during passive viewing suggests the participation of attentional mechanisms and supports this hypothesis^13^,^15^.

### Differences between isolated afferented EVC and deafferented EVC

The contrast between isolated afferented EVC and deafferented EVC is difficult to interpret as it compares two groups with a modified FC. It was primarily intended to be a control for the comparison to the normally afferented visual cortex. In fact, the differences in FC between the isolated afferented central EVC in the RPTV and the deafferented central EVC in the SMD group were roughly similar to the differences observed when compared to the normally afferented central EVC, with the exception of the MOG. The lack of a FC difference in MOG, consistent with the results mentioned above, might reflect the increased adaptive connectivity of both the deafferented and isolated afferented central EVC with this region. The isolated afferented central EVC of RPTV also exhibited a relative increase in FC with the IOG and fusiform gyrus, which are areas of the ventral processing stream.

Compared to the deafferented peripheral EVC in the RPTV patients, the isolated afferented peripheral EVC in the SMD patients exhibited an increased FC with the right sensorimotor cortex, superior temporal gyrus and bilateral LOC. Thus, this unique contrast between afferented and deafferented peripheral EVC also exposes the multisensory connections of the afferented peripheral EVC with sensorimotor and auditory areas^37^,^57^,^58^. Moreover, when seeding from the foot area in SMD patients we found a similar FC pattern, which confirms the multisensory connectivity between afferented peripheral EVC, LOC auditory and somatosensory areas (see Fig. 8).

#### The maps seeded from the S1 areas

The intra-group ROI analyses based on somatosensory seeds validate our resting-state partial correlation approach by showing that the somatosensory topographic system could be mapped in our sighted population as well as in both groups of visually-impaired patients (see Figs 5 and 6). The S1 respective lip and foot areas were perfectly connected between hemispheres in the three groups, see Figs 5 and 6). These results are in line with previous studies showing such a similar organization system in the sighted^40^,^42^. Moreover, the lip area was functionally connected to bilateral inferior frontal gyrus, insula and the right LOC, which is consistent with the literature^52^. We found very minor between-group differences, (see Fig. 7). These results support the claim that observed differences in the visual cortex (Figs 1 to 4) are related to the plastic changes induced by visual field loss.

The analysis seeding the foot sensory projection area also yielded consistent results. The regression of the lip projection area resulted in a negative FC between the foot area and the inferior frontal gyrus and insula. In central visual field loss, the foot area is functionally connected to the isolated afferented peripheral EVC (see Fig. 6c). This coincides with what we found with EVC seeds (see Figs 2b and 8). However, there was no significant difference with the normally sighted (see Fig. 8b).

#### Comparison of the FC between congential, late complete blindness and partial blindness

To the best of our knowledge, few studies^39^,^41^ have compared the rs-FC to retinotopic areas of the blind. Bock *et al*. found that resting-state correlations between V1 and V2/V3 were retinotopically organized in normally sighted, early blind as well as anophthalmic subjects suggesting that retinal waves or visual experience are not necessary to develop a resting-state retinotopic pattern^41^. The team of Striem-Amit seeded both the central and peripheral EVC in congenitally blind subjects and also identified different FC patterns according to the seeds^39^. Interestingly, as compared to sighted controls, they found an increased FC of the peripheral EVC in the congenitally blind group with the same areas that we found in the RP (i.e. IPL/IPS, MFG; see Fig. 4B) and SMD patients (LOC, IOG, ITG, fusiform gyrus; see Fig. 4A). They attributed their results to cross-modal attention plasticity which is consistent with the hypotheses we put forward for our populations. However, these authors found a FC increase of the central EVC with the left inferior frontal gyrus and a FC decrease of both retinotopic areas with the somatosensory and auditory regions. We did not find such results here (see Figs 3, 7 and 8). This might be explained by the extent of visual loss since in a previous study our team showed that the FC pattern of retinotopic regions with Broca’s area differs between RP completely blind and tunnel vision patients^77^. As also shown by the analyses seeding S1 areas (see Figs 5, 6, 7 and 8), certain robust systems (language, auditory, somatosensory) might need profound loss of vision to start developing plasticity^78^,^79^,^80^,^81^.

Overall, the FC pattern of the part of the EVC receiving the remaining visual input supports the first hypothesis that the isolated afferented EVC strengthens the local connections, presumably to tune-up visual information processing. In the deafferented EVC, remodeling concerns more remote connections, and this pattern lends support to the third hypothesis that deafferented EVC likely diverts its processing capacity to higher-order functions that can optimize the residual visual function. The absence of increased local connectivity in the deafferented compared to the normally afferented EVC or its decreased local connectivity when compared to the isolated afferented EVC do not support the view that deafferented EVC rewires the same type of information as the afferented EVC (2^nd^ hypothesis). An alternative explanation for the increased FC of the sensory deprived visual cortex is that it represents noise generated by aberrant autonomous activity^82^ (4^th^ hypothesis). This scenario is unlikely because it would imply strengthened connections between the deafferented EVC and regions to which they are strongly linked in normal vision, an effect that was not confirmed by our data. Rather, the enhanced connectivity of the deafferented primary visual cortex concerned long-range connections involved in higher-order processing. However, as suggested by the thinning of the gray matter in the deafferented EVC in conditions inducing similar visual field defects^83^,^84^,^85^,^86^,^87^,^88^, these long-range connections are not enough to preserve the processing function and structure of the visual areas that lost their sensory input.

## Limitations

Direct implications from the present data on patients’ everyday life and hypotheses about precise cerebral mechanisms cannot be inferred from *rs-*FC data alone. Therefore, the present results and the hypothesis we advanced require further exploration with behavioral and task-based *f*MRI studies.

The best way of designing the ROI for central and peripheral EVC is retinotopy. Unfortunately in central visual loss, the fixation is eccentric and unsteady and as a result, retinotopy would have been particularly challenging and subject to errors. To bypass this limitation, we estimated the central and peripheral EVC by extrapolating the ROI from a retinotopy experiment conducted in a second group of normally sighted subjects. In addition, this method avoids the problem of differences in accuracy when defining EVC across patients and control subjects (see also supplementary Figure S7 for control analysis of localizer definitions). However, since the deafferented primary visual cortex suffers from structural alterations, primarily in the form of gray matter thinning, both in central^83^,^84^,^85^,^86^ and peripheral^83^,^87^ visual field loss, the central and peripheral ROI we employed may not be limited to V1, and overlapped to a certain extent with V2. For this reason we employed the term of “early visual cortex”.

Rs-*f*MRI is subject to reliability problems induced by movement^49^,^89^,^90^. Therefore, we instigated extra measures to control for head movement and to verify that there was no significant difference in head movement across groups (see *pre-processing* section in *Materials and methods* and Supplementary Figures S1 to S4).

It is a convention in the field that increases in functional correlations may reflect increased functional connectivity. However, increases in the BOLD signal can be unrelated to plasticity as demonstrated by higher resting-state firing in blind animals^91^, or enhanced retinotopic rs-signals in anophthalmic individuals possibly due to metabolism alteration^41^. Furthermore, glucose utilization in the human visual cortex is abnormally elevated in early but decreases in late blindness^92^. We have a degree of certainty that the resting-state firing state was equivalent in our three populations since the control analyses seeding S1 areas (see Figs 5, 6, 7 and 8) revealed similar symmetric patterns between hemispheres in all the three groups. Moreover, the selection criteria were stringent to avoid the patients with associated disorders that could account for the metabolic disorders.

Despite numerous claims to the contrary in the literature^28^,^34^,^93^, we cannot exclude that rs-fMRI reflects non neural signals such as blood flow^46^,^94^. It has been shown that the vascular flow from the back to the front of the head is highly similar to the neural eccentric retinotopy^95^. In an attempt to reduce the effect of the vascular component, we used the partial correlation method.

Unlike simple correlation, the partial correlation method has the advantage of identifying false positives when looking for direct connections^53^,^96^,^97^,^98^,^99^,^100^,^101^, but can introduce false negative correlations^48^. Compared to simple correlation analyses (see Supplementary Figures S5 and S6), we found similar negative FC patterns across groups and identified the false positives. Furthermore, we validated the coherence and specificity of the resting-state partial correlation approach through somatosensory seeds (see Figs 5, 6, 7 and 8) and showed that: (1) S1 respective lip and foot areas were perfectly connected between hemispheres as predicted (topographical homologies in the location based on topography) and (2) the somatosensory topographic system in our sighted population was similar to the one described in the literature^40^,^42^ and more importantly, to our visually-impaired subjects (3) analyses seeding EVC and S1 areas were coherent since the FC of S1 to EVC was found after both analyses.

## Conclusion

The current study documented the functional reorganization induced in the primary visual cortex by visual loss specifically affecting either central or peripheral vision. The observed changes suggest two types of adaptive processes. The first involves the afferented parts of the primary visual cortex and engages certain preexisting pathways by enforcing their connections and presumably increasing their processing power to compensate for the loss of the deafferented EVC functions. The second concerns sensory-deprived parts of the primary visual cortex, which strengthens long-range connections, presumably to support high-order mechanisms. These results provide insights into the behavior of EVC functional regions in disease. A greater understanding of these processes is crucial for any attempt to develop efficient rehabilitation strategies or vision restoration.

## Additional Information

**How to cite this article:** Sabbah, N. *et al*. Reorganization of early visual cortex functional connectivity following selective peripheral and central visual loss. *Sci. Rep.* **7**, 43223; doi: 10.1038/srep43223 (2017).

**Publisher's note:** Springer Nature remains neutral with regard to jurisdictional claims in published maps and institutional affiliations.

## Supplementary Material

Supplementary Material

## Figures and Tables

**Table 1:** Subjects’ clinical data.

Subject	Sex	Age	Cause of disease	Age disease onset (first symptoms)	Visual deficit duration
SMD1	F	39	Stargardt macular dystrophy	17	22
SMD2	M	27	Stargardt macular dystrophy	23	4
SMD3	F	47	Stargardt macular dystrophy	18	29
SMD4	M	32	Stargardt macular dystrophy	15	17
SMD5	M	18	Stargardt macular dystrophy	12	6
SMD6	F	56	Stargardt macular dystrophy	24	32
SMD7	F	25	Stargardt macular dystrophy	13	12
SMD8	M	39	Stargardt macular dystrophy	10	29
SMD9	F	40	Stargardt macular dystrophy	16	24
SMD10	M	41	Stargardt macular dystrophy	23	18
SMD11	M	39	Stargardt macular dystrophy	7	32
SMD12	F	58	Stargardt macular dystrophy	17	41
RPTV1	F	43	Retinitis pigmentosa	28	15
RPTV2	H	54	Retinitis pigmentosa	21	33
RPTV3	H	62	Retinitis pigmentosa	39	23
RPTV4	F	37	Retinitis pigmentosa	20	17
RPTV5	F	28	Retinitis pigmentosa	16	12
RPTV6	H	60	Retinitis pigmentosa	4	56
RPTV7	F	61	Retinitis pigmentosa	15	46
RPTV8	H	59	Retinitis pigmentosa	6	53
RPTV9	H	27	Retinitis pigmentosa	4	23
RPTV10	H	29	Retinitis pigmentosa	10	19
RPTV11	F	23	Retinitis pigmentosa	3	20
RPTV12	F	18	Retinitis pigmentosa	1	17
S1	H	31	—	—	—
S2	F	28	—	—	—
S3	F	40	—	—	—
S4	H	59	—	—	—
S5	H	58	—	—	—
S6	F	42	—	—	—
S7	F	54	—	—	—
S8	F	36	—	—	—
S9	H	56	—	—	—
S10	F	54	—	—	—
S11	H	57	—	—	—
S12	H	18	—	——	—
S13	F	24	—	—	—
S14	H	26	—	—	—

SMD: subjects with Stargardt macular dystrophy, RPTV: subjects with retinitis pigmentosa tunnel vision. S: sighted control subjects. Age disease onset: subjects’ age when they experienced the first symptoms of the disease. Visual deficit duration: age minus age at disease onset.

**Table 2:** Peaks of functional connectivity differences between groups.

ROI: Central EVC (regressed peripheral EVC) Contrast Peaks		Brodmann area	Peak X	Peak Y	Peak Z	t value	p value
**Retinitis pigmentosa tunnel vision **> **Normal vision**							
	LH Middle/Superior occipital gyrus	BA 17/18	−23	−92	0	3,40	0,002
	LH Superior temporal gyrus	BA 22	−55	−11	−3	3,31	0,002
	LH Superior temporal gyrus	BA 41	−37	−29	3	4,36	0,000
**Normal vision** > **retinitis pigmentosa tunnel vision**							
	LH Anterior cingulate gyrus	BA 32	−7	34	9	3,34	0,002
	RH Anterior cingulate gyrus	BA 32	8	34	9	2,97	0,005
	RH Anterior middle temporal gyrus	BA 38	41	−2	−21	3,19	0,003
	RH Anterior inferior temporal gyrus	BA 38	35	3	−30	3,53	0,001
	RH Orbital gyrus	BA 11	23	40	6	3,24	0,003
**Stargardt macular dystrophy** > **Normal vision**							
	LH Inferior parietal lobule	BA 39	−40	−49	30	3,43	0,002
	LH Parieto-occipital sulcus/Cuneus	BA 19	−7	−71	24	3,19	0,003
	RH Cuneus	BA 19	8	−69	27	3,68	0,001
	RH Parieto-occipital sulcus/Precuneus	BA 7/BA 19	8	−65	28	3,62	0,001
**Normal vision** > **Stargardt macular dystrophy**							
	LH Orbital gyri	BA 47	−25	39	1	2,79	0,008
	RH Superior temporal sulcus	BA 21	50	−17	−9	3,86	0,000
	RH Middle temporal gyrus	BA 38	50	−8	−15	3,27	0,002
	RH Obital gyri	BA 47	20	40	0	4,57	0,000
**Stargardt macular dystrophy** > **retinitis pigmentosa tunnel vision**							
	LH Superior occipital gyrus	BA 18	−22	−89	27	3,68	0,001
	LH Parieto-occipital sulcus	BA 19	−7	−68	30	2,51	0,017
	RH Inferior parietal lobule	BA 19	47	−56	30	3,00	0,005
	RH Parieto-occipital sulcus/Cuneus	BA 19	5	−77	30	3,19	0,003
	RH Precuneus	BA 7	8	−70	34	2,85	0,007
**retinitis pigmentosa tunnel vision** > **Stargardt macular dystrophy**							
	LH Fusiform gyrus/Collateral sulcus	BA 37	−28	−56	−12	3,25	0,003
	LH Inferior occipital gyrus	BA 18	−40	−80	−12	3,39	0,002
	LH Supramarginal gyrus	BA 40	−49	−20	18	3,85	0,000
ROI: Peripheral EVC (regressed central EVC)							
**retinitis pigmentosa tunnel vision** > **Normal vision**							
	RH Inferior parietal lobule	BA 39	56	−50	36	3,61	0,001
	RH Posterior middle frontal gyrus	BA 6	44	1	42	4,48	0,000
	RH Anterior middle frontal gyrus	BA 44	35	28	33	3,01	0,005
	RH Superior frontal gyrus (BA 9)	BA 9	11	31	48	3,48	0,001
**Normal vision** > **retinitis pigmentosa tunnel vision**							
	LH Middle occipital gyrus	BA 18	−28	−89	−6	3,75	0,001
	LH Superior occipital gyrus	BA 18	−25	−89	3	3,05	0,004
	LH Middle temporal gyrus	BA 21	−55	−17	0	3,91	0,000
	LH Post central gyrus	BA 2	−13	−38	51	4,20	0,000
	RH Inferior occipital gyrus	BA 18	33	−86	−18	3,04	0,004
	RH Middle occipital gyrus	BA 18	26	−80	3	4,24	0,000
	RH Superior occipital gyrus	BA 18/19	20	−80	21	3,19	0,003
	RH medial central sulcus/medial post and precentral gyri	—	8	−41	59	3,26	0,002
**Stargardt macular dystrophy** > **Normal vision**							
	LH Lingual gyrus	BA 19	−22	−65	−9	2,91	0,006
	RH Lingual gyrus	BA 19	17	−50	−6	3,26	0,002
	RH Fusiform gyrus	BA 20	47	−38	−15	4,54	0,000
	RH parahippocampal gyrus	BA 35	35	−31	−21	3,10	0,004
	RH Inferior temporal gyrus	BA 20	55	−42	−11	2,53	0,016
	RH Inferior occipital gyrus	BA 18	44	−68	−12	3,04	0,004
	RH Inferior temporal gyrus	BA 20	53	−47	−9	3,57	0,001
	RH Inferior temporal sulcus	BA 19	35	−68	6	3,42	0,002
	RH Orbital gyrus	BA 11	14	40	−3	3,49	0,001
**ROI: Peripheral EVC (regressed central EVC) Constrast peaks**							
**Normal vision** > **Stargardt macular dystrophy**							
	LH Anterior cingulate gyrus	BA 24	−10	22	30	3,03	0,005
	LH Cuneus	BA 18	−7	−71	27	3,26	0,002
	RH Cuneus/Parieto-occipital sulcus	BA 18	8	−65	27	4,05	0,000
	RH Pre-cuneus	BA 7	8	−65	27	4,05	0,000
**Stargardt macular dystrophy** > **retinitis pigmentosa tunnel vision**							
	LH Lingual gyrus/Collateral sulcus	BA 19	−25	−62	−15	4,34	0,000
	LH Fusiform gyrus	BA 19	−33	−68	−15	3,79	0,001
	LH Inferior occipital gyrus	BA 18	−43	−80	−18	5,34	0,000
	LH Middle occipital gyrus	BA 19/19	−35	−86	−3	3,50	0,001
	LH Central sulcus (dorsal)	BA 1/BA 4	−10	−35	57	3,21	0,003
	LH Medial precentral gyrus	BA 4	0	−32	59	3,47	0,001
	RH Lingual gyrus/Collateral sulcus	BA 19	23	−53	−6	3,81	0,001
	RH Inferior occipital gyrus	BA 19	33	−65	−17	3,19	0,003
	RH Fusiform gyrus	BA 37	38	−47	−18	4,25	0,000
	RH Parahippocampal gyrus	BA 35	26	−29	−21	3,76	0,001
	RH Inferior temporal gyrus (posterior)	BA 20	46	−50	−15	2,86	0,007
	RH Inferior occipital gyrus	BA 18/19	38	−68	−15	4,59	0,000
	RH Middle occipital gyrus	BA 18	32	−71	6	4,59	0,000
	RH Superior occipital gyrus	BA 18	26	−80	16	4,07	0,000
	RH Middle occipital gyrus/Middle temporal gyrus	BA 19	41	−63	6	4,04	0,000
	RH Superior temporal gyrus/Superior temporal sulcus	BA 21/22	50	−14	−6	3,80	0,001
	RH Superior parietal lobule/Post central sulcus	BA 5	26	−38	45	3,61	0,001
	RH Post central gyrus/Central sulcus	BA 2	29	−30	54	3,03	0,005
	RH Precentral gyrus	BA 4	20	−17	54	3,09	0,004
**retinitis pigmentosa tunnel vision** > **Stargardt macular dystrophy**							
	LH Cuneus	BA 19	−7	−71	27	−3,77	0,001
	LH Anterior cingulate	BA 32	−4	22	24	−3,42	0,002
	LH Middle frontal gyrus	BA 46	−28	40	21	−3,76	0,001
	RH Inferior parietal lobule	BA 39	50	−59	30	−2,78	0,009
	RH Middle frontal gyrus	BA 9	35	25	36	−4,46	0,000
	RH Superior frontal gyrus	BA 9	14	31	45	−2,67	0,011
	RH Cuneus	BA 19	11	−71	27	−3,63	0,001

ROI: region-of-interest. Peaks x, y, z: Talairach coordinates. P values corrected for multiple comparisons. LH: Left hemisphere. RH: Right hemisphere. BA: Brodmann areas.
