# Supplementary material for: Reorganization of early visual cortex functional connectivity following selective peripheral and central visual loss
Source: Sci Rep. 2017 Feb 24;7:43223. doi: 10.1038/srep43223 (PMC5324137; doi:10.1038/srep43223)
Supplement: Supplementary Material [file srep43223-s1.pdf]

Title

Reorganization of early visual cortex functional connectivity following selective peripheral and central visual loss

Authors

Norman Sabbah,*[1-4] Nicolae Sanda,*[1-5] Colas N. Authié,[1-4] Saddek Mohand-Saïd,[1-4] José-Alain Sahel,[1-4,7-8] Christophe Habas,[1-3,6] Amir Amedi,[1-3,10-12] Avinoam B. Safran,[1-4,9]

Supplementary material

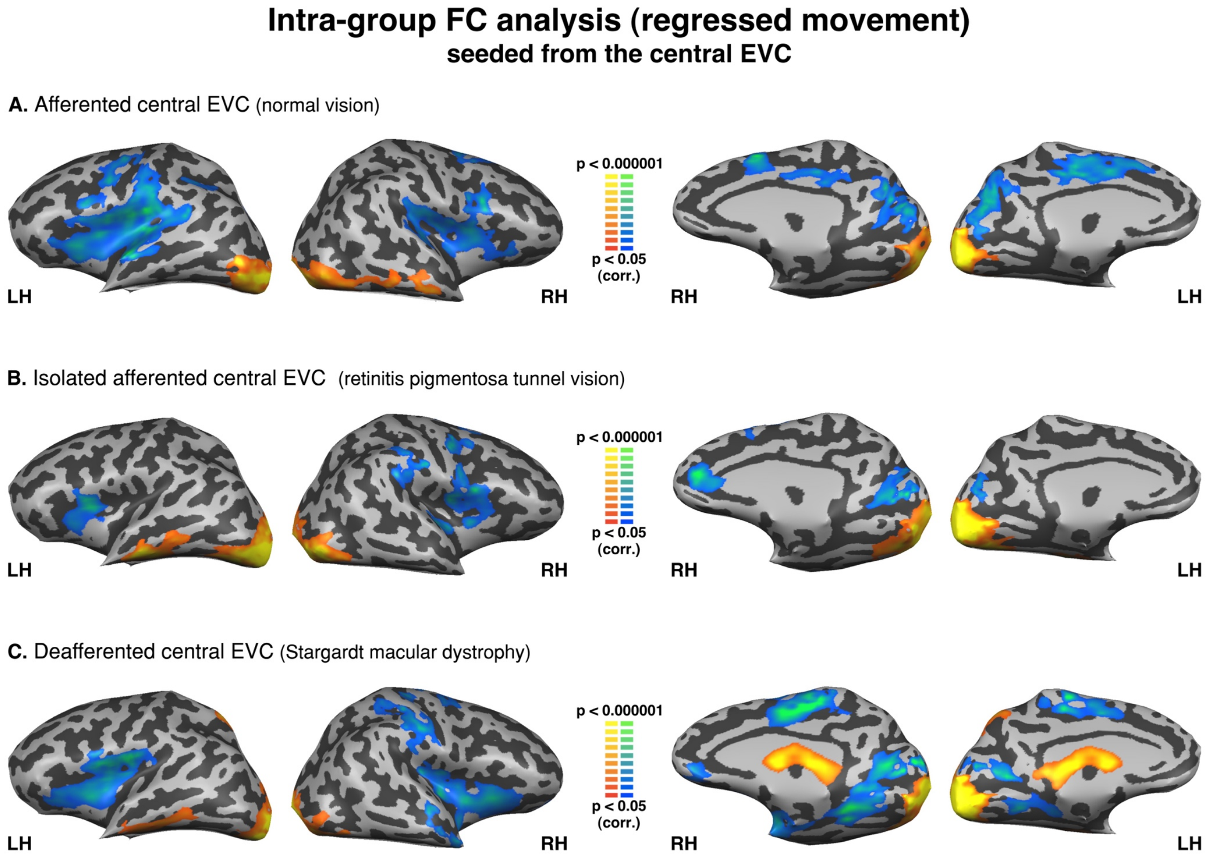

Figure S1. Intra-group analysis of functional connectivity seeded from the central EVC (peripheral EVC regressed and movement regressed). *The maps are shown in mesh for the* ***(A)*** *afferented central EVC (normally sighted)* ***(B)*** *isolated afferented central EVC (retinitis pigmentosa tunnel vision)* ***(C)*** *deafferented central EVC (Stargardt macular dystrophy). Yellow-orange depicts areas of higher positive connectivity for each group, and green-blue higher negative connectivity. LH: left hemisphere, RH: right hemisphere.*

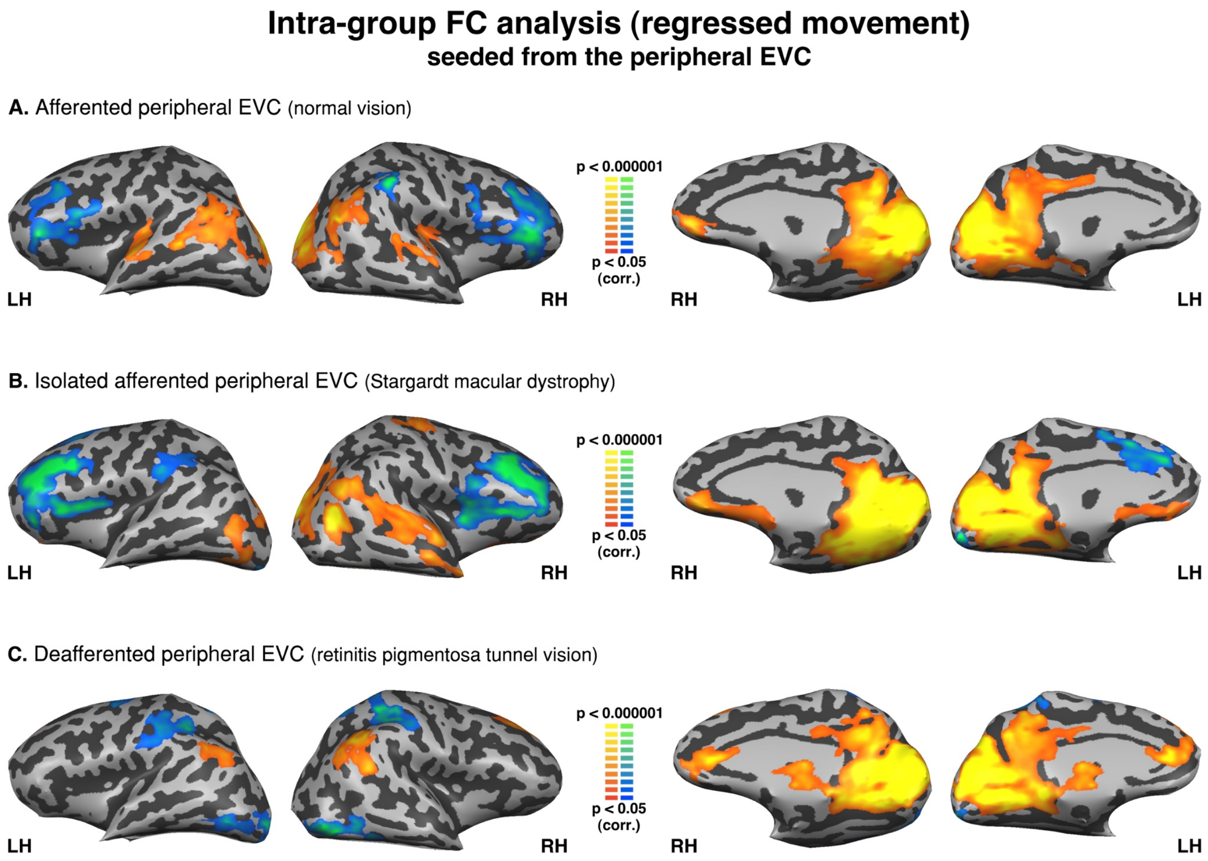

Figure S2. Intra-group analysis of functional connectivity seeded from the peripheral EVC (central EVC and movement regressed). *The maps are shown in mesh for the* ***(A)*** *afferented peripheral EVC (normally sighted); * the dotted line on the median aspect of the brain represents the seed region (peripheral EVC)* ***(B)*** *isolated afferented peripheral EVC (Stargardt macular dystrophy)* ***(C)*** *deafferented peripheral EVC (retinitis pigmentosa tunnel vision). Yellow-orange depicts areas of higher positive connectivity for each group, and green-blue higher negative connectivity. LH: left hemisphere, RH: right hemisphere.*

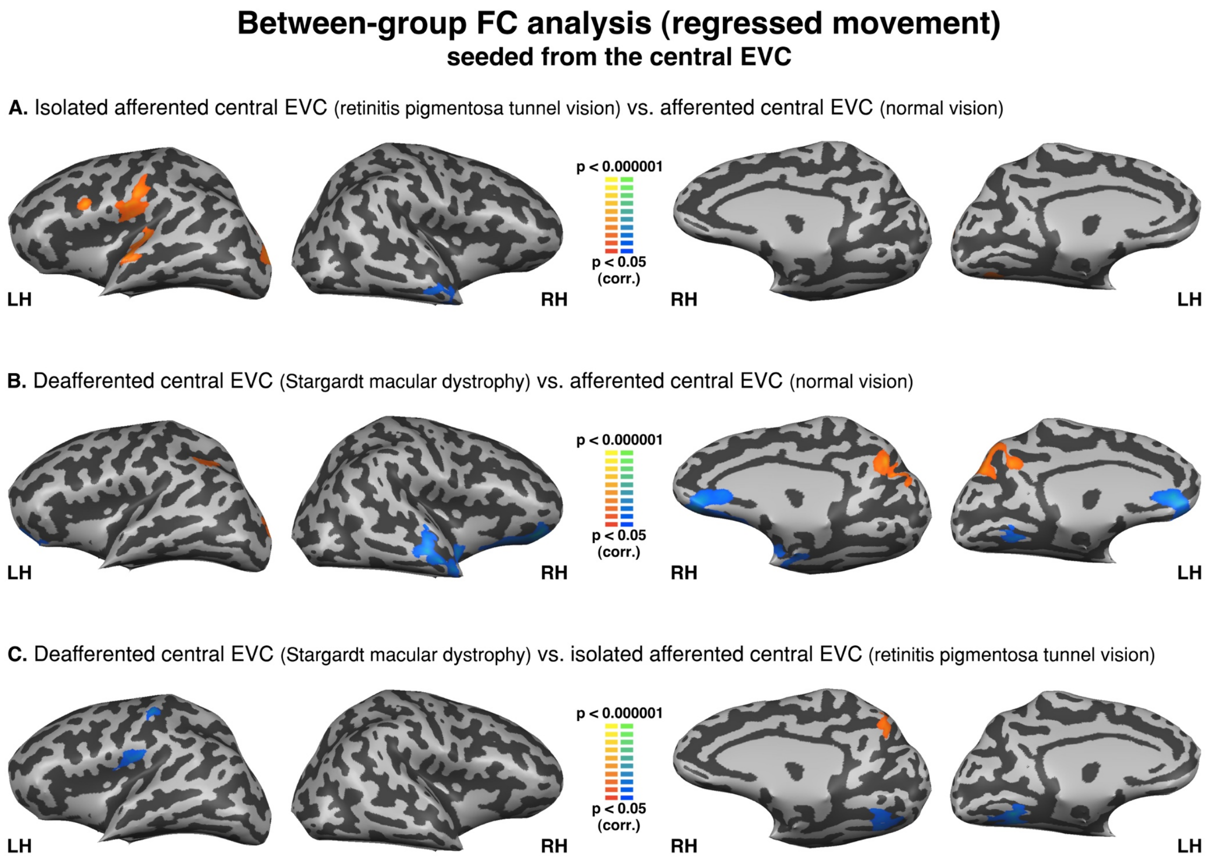

Figure S3. Between-group analysis of functional connectivity seeded from the central EVC (peripheral EVC and movement regressed). *The maps are shown in mesh for* ***(A)*** *retinitis pigmentosa tunnel vision vs. normal vision* ***(B)*** *Stargardt macular dystrophy vs. normal vision* ***(C)*** *Stargardt macular dystrophy vs. retinitis pigmentosa tunnel vision (yellow-orange depicts areas of higher positive/lower negative functional connectivity with the central EVC for the first group compared to the second, and green-blue the opposite comparison; LH: left hemisphere, RH: right hemisphere.*

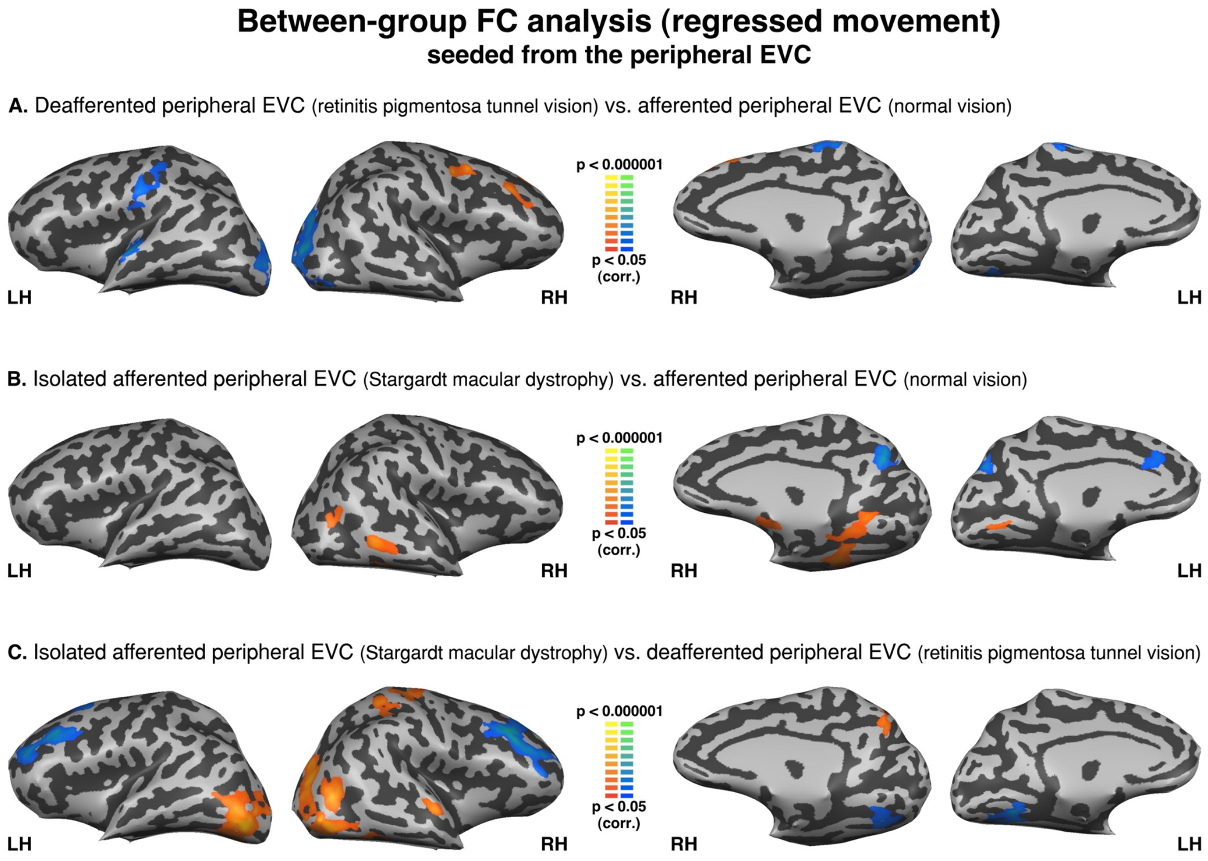

Figure S4. Between-group analysis of functional connectivity seeded from the peripheral EVC (central EVC and movement regressed). *The maps are shown in mesh for* ***(A)*** *retinitis pigmentosa tunnel vision vs. normal vision* ***(B)*** *Stargardt macular dystrophy vs. normal vision* ***(C)*** *Stargardt macular dystrophy vs. retinitis pigmentosa tunnel vision (yellow-orange depicts areas of higher positive/lower negative functional connectivity with central EVC for the first group compared to the second, and green-blue the opposite comparison; LH: left hemisphere, RH: right hemisphere).*

Comment to supplementary Figures S1, S2, S3, S4:

The maps are quite similar to the ones presented in main **Figures 1 to 4** using the motion correction implemented during the pre-processing stages.

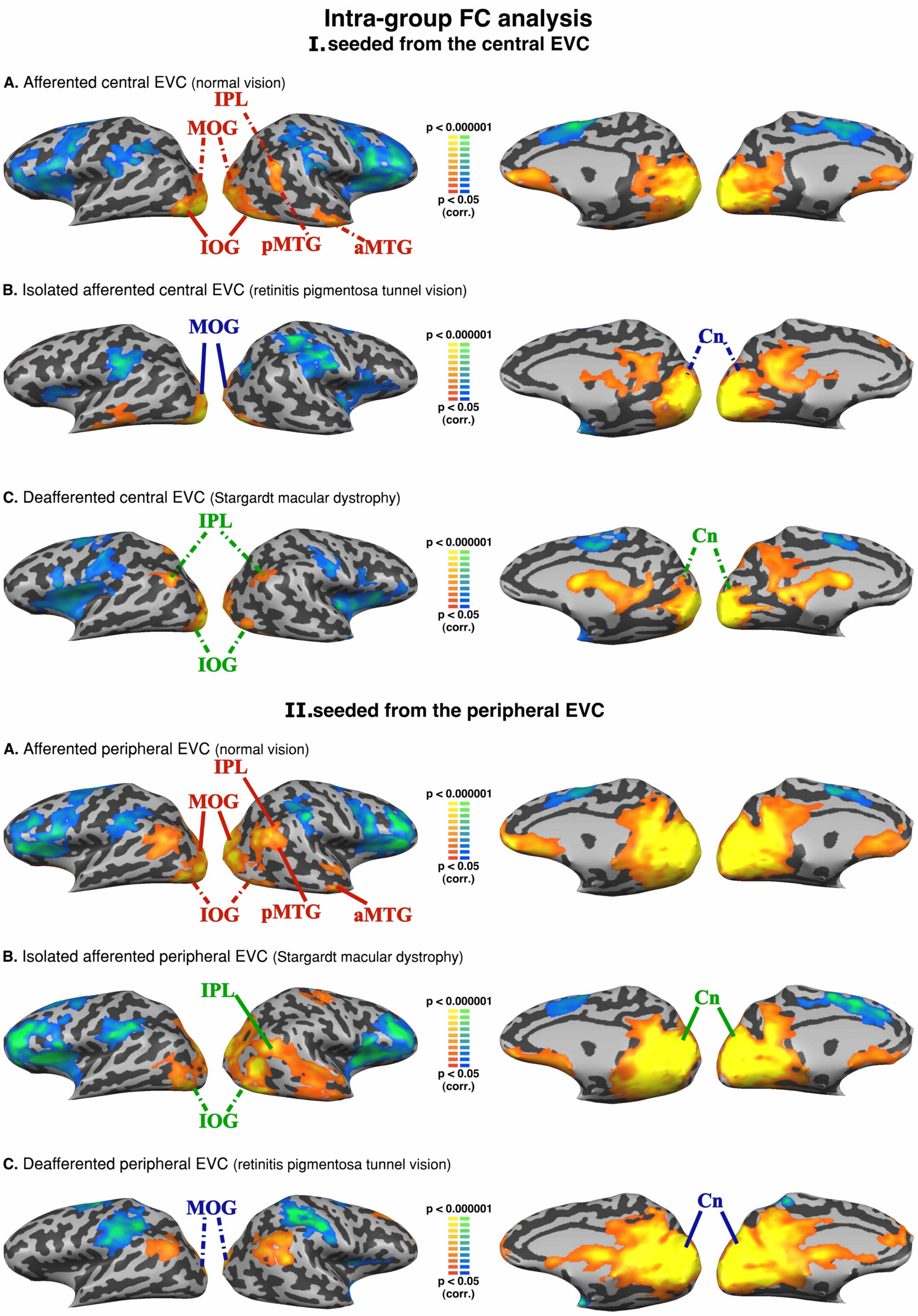
Intra-group FC analysis
I. seeded from the central EVC
A. Afferented central EVC (normal vision)
IPL
MOG
IOG
pMTG
aMTG
p < 0.000001
p < 0.05 (corr.)
B. Isolated afferented central EVC (retinitis pigmentosa tunnel vision)
MOG
Cn
C. Deafferented central EVC (Stargardt macular dystrophy)
IPL
IOG
Cn
II. seeded from the peripheral EVC
A. Afferented peripheral EVC (normal vision)
IPL
MOG
IOG
pMTG
aMTG
B. Isolated afferented peripheral EVC (Stargardt macular dystrophy)
IPL
IOG
Cn
C. Deafferented peripheral EVC (retinitis pigmentosa tunnel vision)
MOG
Cn

Figure S5. Intra-group analysis of functional connectivity seeded from I. The central and II. The peripheral EVC (without regression). The maps are shown in mesh. Yellow-orange depicts areas of higher positive functional connectivity for each group, and green-blue higher negative functional connectivity. I. The rs-FC seeded from the central EVC: **(A)** the afferented central EVC (normal vision) **(B)** the isolated afferented central EVC (retinitis pigmentosa tunnel vision) **(C)** the deafferented central EVC (Stargardt macular dystrophy) **II.** The rs-FC seeded from the peripheral EVC: **(A)** the afferented central EVC (normal vision) **(B)** the isolated afferented central EVC (retinitis pigmentosa tunnel vision) **(C)** deafferented central EVC (Stargardt macular dystrophy). Yellow-orange depicts areas of higher positive functional connectivity for each group, and green-blue higher negative functional connectivity. Dashed lines: probable false positive correlations; solid lines: the likely origin of the positive correlations. Red lines: areas that differ with vs. without the partial correlation method in normal vision. Green lines: areas that differ with vs. without the partial correlation method in Stargardt macular dystrophy. Blue lines: areas that differ with vs. without the partial correlation method in retinitis pigmentosa tunnel vision (see also **Figure 1**). Cn: cuneus; IOG: inferior occipital gyrus; IPL: inferior parietal lobule; MTS: middle temporal sulcus; MOG: middle occipital gyrus. pMTG: posterior middle temporal gyrus. aMTG: anterior middle temporal gyrus.

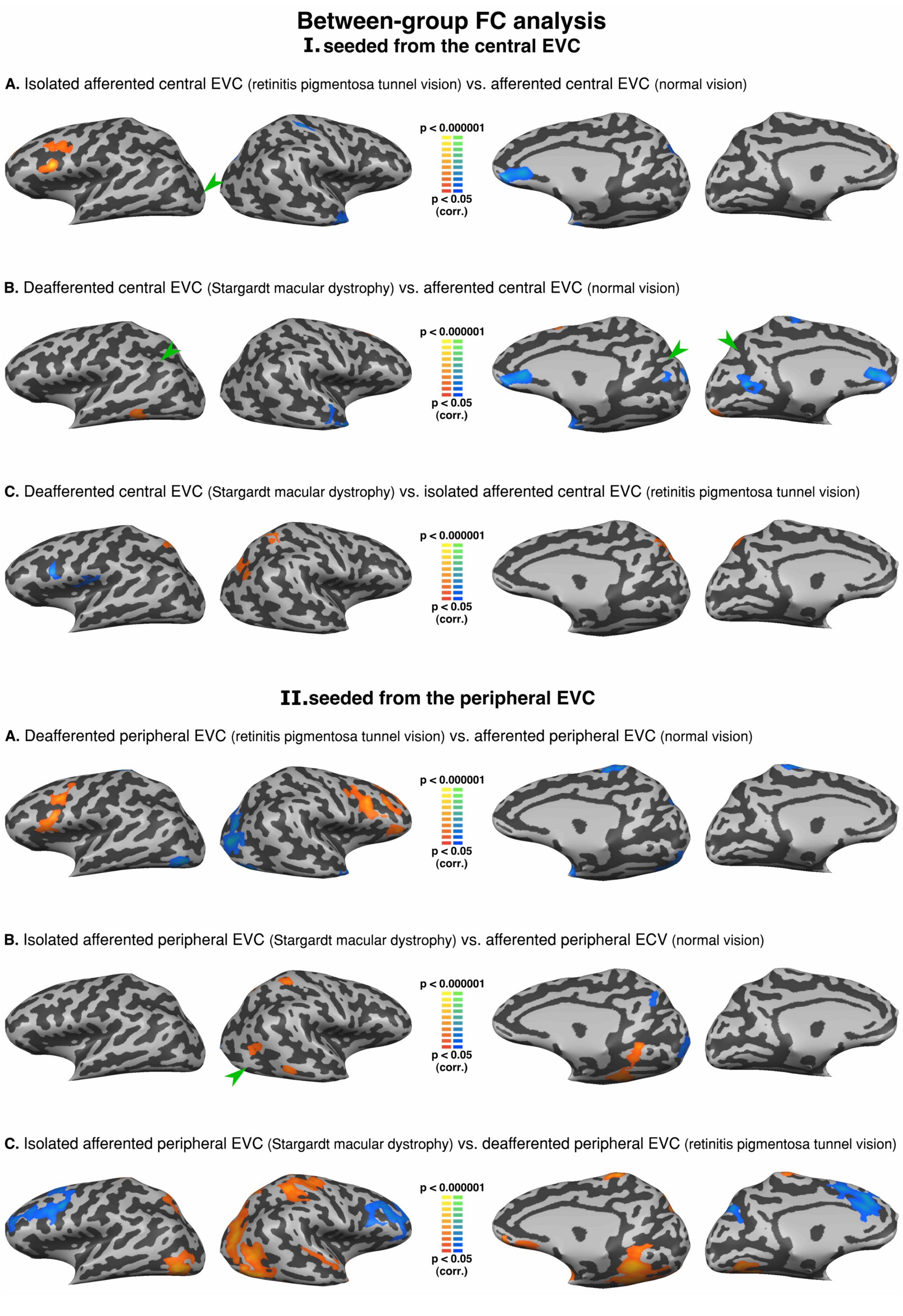

Figure S6. Between-group analysis of functional connectivity seeded from I. The central and II. The

peripheral EVC (without regression). The maps are shown in mesh. Yellow-orange depicts areas of higher positive/lower negative functional connectivity with **I.** The central EVC or **II.** The peripheral EVC for the first group compared to the second, and green-blue the opposite comparison. **I.** Seeded from the central EVC: **(A)** retinitis pigmentosa tunnel vision vs. normal vision **(B)** Stargardt macular dystrophy vs. normal vision **(C)** Stargardt macular dystrophy vs. retinitis pigmentosa tunnel vision. **II.** seeded from the peripheral EVC: **(A)** retinitis pigmentosa tunnel vision vs. normal vision **(B)** Stargardt macular dystrophy vs. normal vision **(C)** Stargardt macular dystrophy vs. retinitis pigmentosa tunnel vision. Green arrowheads show lack of differences in rs-FC compared to the partial correlation analysis.

Comment to Supplementary Figures S5, S6:

The partial correlation method has the advantage of being able to exclude false positives, which was why it was chosen in the current study. Supplementary **Figures S5** and **S6** present the intra-group analysis for central and peripheral EVC as well as the intragroup comparisons without partial correlation. **Figure S5** depicts certain regions that are likely false positive correlations and we point to their probable origin. These false positive rs-FC may be the cause of the differences in FC between groups as illustrated by the green arrowheads in Figure S6. In addition, at the intra-group level, the negative FC to both central and peripheral EVC is fairly similar with (see **Figures 1 and 2**) or without (**Figure S5**) calculating by the partial correlation method.

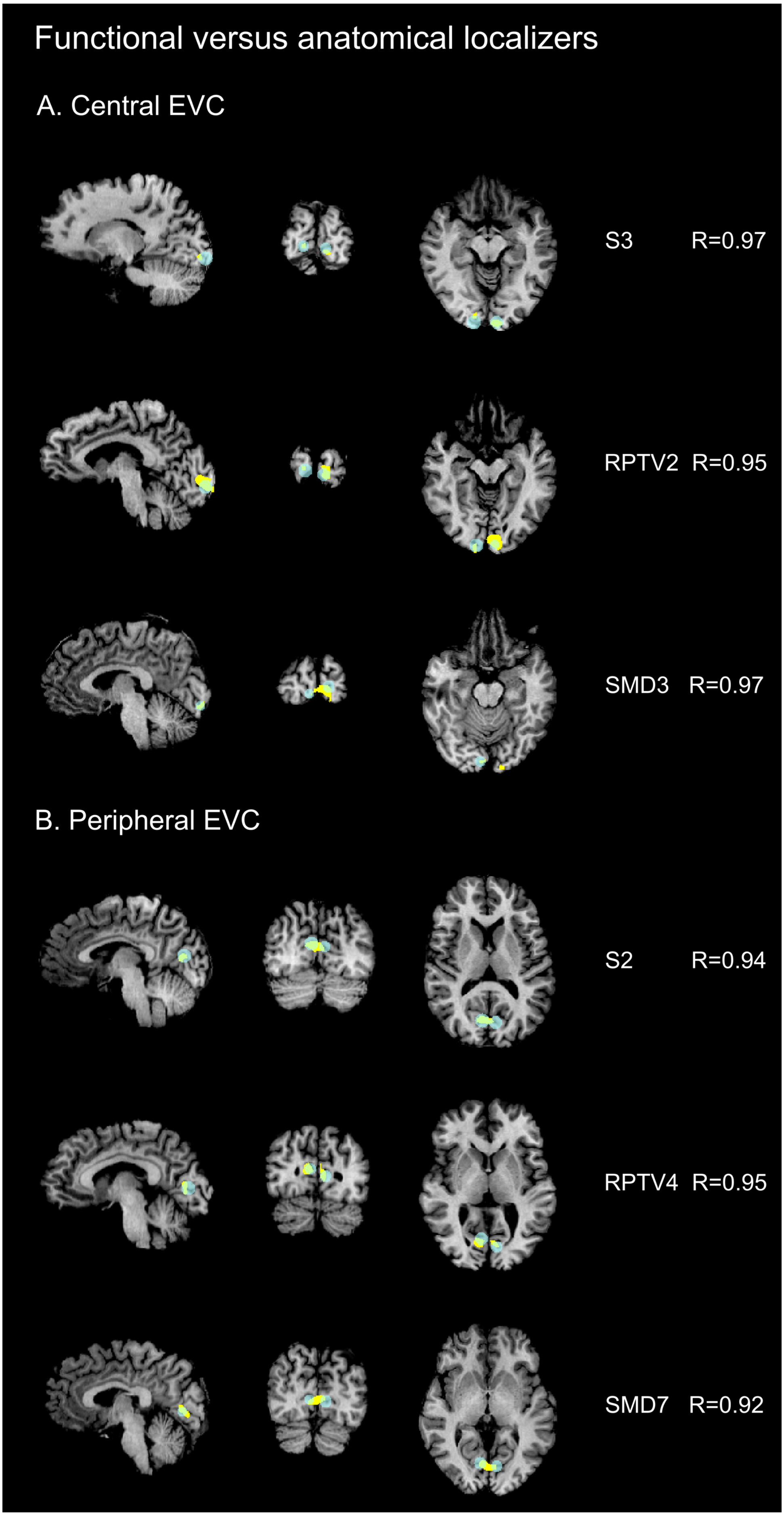
Functional versus anatomical localizers
A. Central EVC
S3 R=0.97
RPTV2 R=0.95
SMD3 R=0.97
B. Peripheral EVC
S2 R=0.94
RPTV4 R=0.95
SMD7 R=0.92

Figure S7. Correlation between functional and anatomical localizers at the individual level. Two individual subjects were randomly selected from each group. *For each individual subject, Pearson correlation tests (R) were run to test for time courses correlation between seed regions defined based on external functional localizers and seed regions defined based on anatomical localizers (as defined by 7-mm spheres on A. posterior or B. anterior portions of the calcarine sulcus). Yellow depicts areas defined by the functional localizers whereas blue depicts areas defined by the anatomical localizers on the fMRI slices from each individual. S: sighted control subjects, RPTV: subjects with retinitis pigmentosa tunnel vision, SMD: subjects with Stargardt macular dystrophy.*

The control analysis of the localizer definitions revealed a strong correlation between the time courses of seed regions defined based on external functional localizers and seed regions defined based on anatomical localizers for each individual, regardless of group.
